# PREventing Maternal And Neonatal Deaths (PREMAND): a study protocol for examining social and cultural factors contributing to infant and maternal deaths and near-misses in rural northern Ghana

**DOI:** 10.1186/s12978-016-0142-z

**Published:** 2016-03-09

**Authors:** Cheryl A. Moyer, Raymond A. Aborigo, Elizabeth B. Kaselitz, Mira L. Gupta, Abraham Oduro, John Williams

**Affiliations:** University of Michigan Medical School, 1111 Catherine St, Ann Arbor, MI 48109 USA; Navrongo Health Research Centre, PO Box 114, Navrongo, UE/R Ghana

**Keywords:** Social autopsy, Near misses, Neonatal mortality, Maternal mortality, Africa, Developing countries, Low- and middle-income countries, Geospatial mapping, Local health innovations, Community engagement

## Abstract

**Plain English Summary:**

The Preventing Maternal And Neonatal Deaths (PREMAND) project works to understand the social and cultural factors that may contribute to the deaths and near-misses (people who almost die but end up surviving) of mothers and babies in four districts in Northern Ghana. Examples of these factors include such thing as treating a sick baby at home with traditional medicine instead of going to a hospital or health center, or pregnant women needing permission from several people before they can go to a hospital to deliver. These social and cultural factors will be placed on a map to understand where patterns and clusters of deaths and near-misses are present in these four communities. The final phase of the project will include support and small grants for community members and local leaders to use these maps and this information to create their own solutions that address the specific needs of each community.

**Abstract:**

**Background**

While Ghana is a leader in some health indicators among West African nations, it still struggles with high maternal and neonatal morbidity and mortality rates, especially in the northern areas. The clinical causes of mortality and morbidity are relatively well understood in Ghana, but little is known about the impact of social and cultural factors on maternal and neonatal outcomes. Less still is understood about how such factors may vary by geographic location, and how such variability may inform locally-tailored solutions.

**Methods/Design**

Preventing Maternal And Neonatal Deaths (PREMAND) is a three-year, three-phase project that takes place in four districts in the Upper East, Upper West, and Northern Regions of Ghana. PREMAND will prospectively identify all maternal and neonatal deaths and ‘near-misses’, or those mothers and babies who survive a life threatening complication, in the project districts. Each event will be followed by either a social autopsy (in the case of deaths) or a sociocultural audit (in the case of near-misses). Geospatial technology will be used to visualize the variability in outcomes as well as the social, cultural, and clinical predictors of those outcomes. Data from PREMAND will be used to generate maps for local leaders, community members and Government of Ghana to identify priority areas for intervention. PREMAND is an effort of the Navrongo Health Research Centre and the University of Michigan Medical School.

**Discussion**

PREMAND uses an innovative, multifaceted approach to better understand and address neonatal and maternal morbidity and mortality in northern Ghana. It will provide unprecedented access to information on the social and cultural factors that contribute to deaths and near-misses in the project regions, and will allow such causal factors to be situated geographically. PREMAND will create the opportunity for local, regional, and national stakeholders to see how these events cluster, and place them relative to traditional healer compounds, health facilities, and other important geographic markers. Finally, PREMAND will enable local communities to generate their own solutions to maternal and neonatal morbidity and mortality, an effort that has great potential for long-term impact.

**Electronic supplementary material:**

The online version of this article (doi:10.1186/s12978-016-0142-z) contains supplementary material, which is available to authorized users.

## Background

Pregnancy and childbirth pose a significant risk to mothers and babies in low- and middle-income countries (LMICs). Nearly 300,000 women die each year from pregnancy-related causes, and 3 million babies die within a month of being born [1, 2]. While Ghana has better health indicators than many of its West African neighbors, it faces significant challenges in improving maternal and newborn health [3]. An average of 25 out of every 1000 babies born each year in Ghana do not survive their first month, compared to four out of 1000 in the United States [4]. Yet such rates vary widely by region and even by district within Ghana. For example, two adjacent districts in northern Ghana have radically different neonatal mortality rates: the Kassena-Nankana Districts report a rate of 19 per 1000 live births, compared to nearly 60 per 1000 live births in Sissala-East [5, 6].

The clinical causes of both maternal and neonatal mortality are well known. Globally, the leading causes of maternal deaths are hemorrhage, hypertensive disorders, sepsis and obstructed labor, accounting for approximately 60 % of all maternal deaths [7]. The leading causes of death among newborns include complications from prematurity, asphyxia, and severe infections, accounting for 74 % of all neonatal deaths worldwide [1, 8, 9]. Such conditions, if treated rapidly and appropriately by knowledgeable healthcare providers, do not have to result in death. Thus mothers and babies are often dying of largely preventable causes.

The clinical cause of death is only one piece of the puzzle: often there are significant social and cultural factors that influence illness, symptom recognition, and care seeking. Local preferences for in-home births [10] may encourage women to use traditional birth attendants, for example, limiting access to emergency obstetric care in the event of complications and putting both women and their babies at higher risk of infection due to non-sterile delivery [11]. Families and communities also play a role in the recognition of infant illnesses, as well as influencing the timing and type of treatment sought [12]. For example, one traditional remedy for neonatal asphyxia in northern Ghana is to hit a spoon against a cup next to the baby’s ear to encourage it to breathe, rather than seeking care at a local clinic [12]. Other factors such as cost, accessibility, the reputation of local health facilities, and family and community hierarchies requiring women to seek approval before going to a facility may influence care seeking, and in turn affect outcomes [10].

The issues are similar for situations in which mothers and babies may get very sick, but survive. These situations, termed “near-misses”, are also not well understood and are rarely documented [13–15]. What are the factors that led up to their illness, and what were the factors that allowed them to survive? As is the case with deaths, understanding the social and cultural factors that influence near-misses is likely to vastly enhance the ability of communities, governments, and programs to prevent maternal and infant deaths in the future.

Understanding the social and cultural factors that contribute to maternal and infant deaths is a critically important factor in addressing maternal and neonatal mortality. In recent years, a new technique called “Social Autopsy” has emerged that explores the social and community factors that precede and contribute to maternal and neonatal mortality [16, 17]. Social Autopsies produce a richer and more substantive understanding of the context of deaths through an interview process with family and community members able to comment on the social, cultural, and behavioral factors that may have impacted maternal and neonatal outcomes. Unlike verbal autopsies that assist in pinpointing the biomedical cause of death and were developed in large part by clinicians, social autopsy examines the social, cultural, and health systems contributors. Our study will utilize both verbal and social autopsy techniques to understand the clinical cause of death, as well as the social and cultural context surrounding each death.

We believe a similar method can be applied to “near-miss” situations where a death was narrowly averted to illustrate the factors that may have contributed to survival. Building on existing models [16, 17], we have adapted and tailored screening tools to capture and assess the social and cultural contributors to neonatal and maternal near-misses (see Table 1 for a description of the Maternal Sociocultural Assessment (MSCA) and the Neonatal Sociocultural Assessment (NSCA)).

**Table 1:** PREMAND project screening tools

Screening Tool	Description
Short form RAMOS (Reproductive Aged Mortality Survey) for identifying potential maternal deaths	This is an instrument based on previous work by Geynisman et al. (2011) that demonstrated the use of a subset of the 39-question Reproductive Age Mortality Survey was positively predictive in identifying deaths that were ultimately classified as maternal mortality deaths [28]. The point of this screening tool is to reduce the number of verbal and social autopsies that end up not being cases of maternal mortality, but instead are simply deaths among women of reproductive age. Field workers will train CKIs to administer the RAMOS screening tool whenever they learn of the death if a woman of reproductive age in the community. The questions on the RAMOS screening tool are: o Was the woman recently pregnant? (Within the past 6 weeks?) o Was she pregnant when she died? o Was she bleeding from the vagina? o Do you think pregnancy had anything to do with her death?
Neonatal Screening Tool for identifying potential neonatal deaths	This is a short instrument to determine if the death of an infant qualifies as a neonatal death. The instrument asks if there was a baby who died, and if yes, did the baby die within 28 days (4 weeks) of its birth.
Maternal Near-Miss Screening Tool for identifying maternal near-misses	This is a checklist developed by the World Health Organization [23] and initially modified for use in Ghana by Tuncalp et al. (2013) for a study of maternal near-misses at Korle Bu Teaching Hospital in Accra [29]. The instrument has been subsequently modified in collaboration with a team of post-doctoral researchers from Ghana led by one of the Co-Directors (CM) of this project for a study of near-misses in the southern part of Ghana conducted during the dry season of 2015 at the Korle Bu Teaching Hospital, the Komfo Anokye Teaching Hospital, and the Cape Coast Regional Hospital (the project is known as MISS– the Maternal and Infant Survival Study). This instrument will allow providers to complete a brief checklist to determine if a woman can be classified as a “near-miss” based upon the answers to a few critical clinical questions.
Neonatal Near-Miss Screening Tool for identifying neonatal near-misses	This is a checklist that has been developed in collaboration with a team of post-doctoral researchers from Ghana led by one of the Co-Directors (CM) of this project for a study of near-misses in the southern part of Ghana conducted in the Spring of 2015 at Korle Bu Teaching Hospital, Komfo Anokye Teaching Hospital, and Cape Coast Regional Hospital (the MISS project, described above). The instrument was developed based on existing neonatal morbidity measures [30] and modeled after the WHO Maternal Near-Miss criteria. The instrument will allow providers to complete a brief checklist to determine if a baby can be classified as a ‘near-miss’ based upon the answers to a few critical clinical questions.

Evidence from Ghana and elsewhere suggests that the social, cultural, and health system contributors to maternal and neonatal morbidity and mortality may vary by location [18–20]. Variability is not only seen across nations, but also within countries and even within regions and districts. Yet without understanding the extent of this variability, it is not possible to develop effective, locally-tailored interventions.

This project, Preventing Maternal And Neonatal Death (PREMAND), aims not only to implement social autopsy and near-miss assessments in rural northern Ghana, but to use geospatial technology to visualize the variability in outcomes as well as the predictors of those outcomes. Ultimately, data from PREMAND will be used to generate maps that can be used by local leaders and community members to identify priority areas for intervention. The project is guided by the following three objectives:

1. To improve understanding of maternal and neonatal morbidity and mortality through the implementation of social autopsy and near-miss assessments;
2. To utilize geographic technology to combine the identified trends contributing to maternal and neonatal deaths and near-misses with locational data to make the results more actionable for communities, government leaders, and the donor community; and
3. To engage community members in programming tailored to address the challenges featured in their specific maternal and neonatal profile, as a means to locally sourcing and testing potential programmatic responses

## Methods

PREMAND is a three-year, three-phase project funded by the Ghana Mission of the United States Agency for International Development (USAID-Ghana), with additional funding from the Navrongo Health Research Centre and the University of Michigan. PREMAND includes primary data collection in four districts of northern Ghana, the development of an interactive mapping interface, and the identification of selected communities within the project districts to serve as “innovation sites” where locally-driven pilot projects will be launched in response to PREMAND findings.

### Project sites

**Fig. 1:**
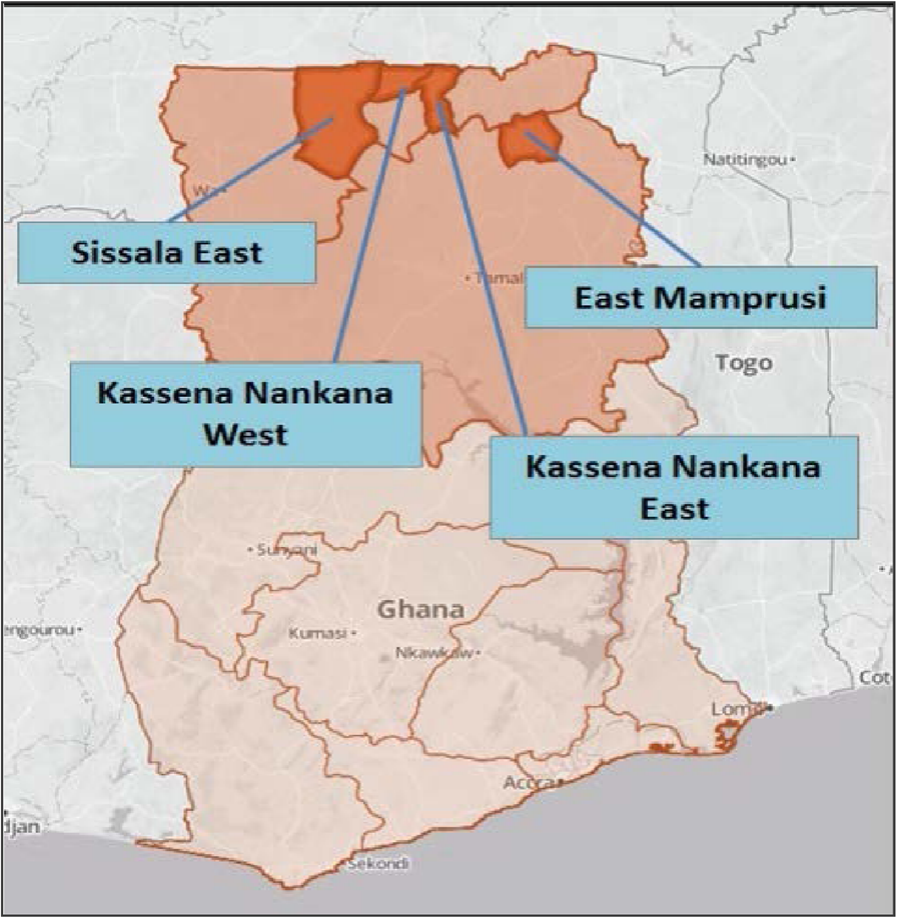
Project districts

Four districts across the Upper West, Upper East, and Northern Regions of Ghana have been selected as the project zones: Sissala East, Kassena-Nankana East, Kassena-Nankana West, and East Mamprusi. Figure 1 illustrates the project districts. These districts were selected based upon several criteria. First, the Kassena-Nankana Districts (KNDs) are the home of the Navrongo Health Research Centre (NHRC), one of the central partners in this endeavor. NHRC is one of three research outposts of the Ghana Health Service and has been running a demographic surveillance site with ongoing population monitoring for more than 25 years [21, 22]. Launching a project in the KNDs will allow us to work through the methodological challenges of implementation with a seasoned team in a well-known location. The two additional districts were selected to reflect more challenging maternal and neonatal statistics than in the KNDs, while still being close enough to NHRC to maximize feasibility. Sissala East, for example, is contiguous with KND West. West Mamprusi was our initial choice for the fourth district, yet the presence of the Millenium Villages Project in West Mamprusi increased the likelihood of contamination for both our project and theirs. Given proximity to KND, East Mamprusi appeared to be the next logical choice as a district with challenging health indicators in the Northern Region that was feasible for NHRC to focus upon.

### Study participants

#### Social autopsy participants

In the case of maternal and neonatal deaths, this project will involve identifying women who died during or shortly after pregnancy, as well as babies who died within 28 days of their birth. Deaths will be identified by community volunteers and health care providers who will be trained to pose four screening questions for each maternal death and one screening question for each infant death (See Table 1). For each identified death, we will seek to speak with the closest living relative or care provider who was with the mother or with the baby before death. Thus, participants must have been a relative or care provider for a woman whose death was associated with her pregnancy, or for a baby who died in the first 28 days of life. Participants must speak one of the following five languages: Mampruli, Sissali, Kasem, Nankam, or English.

#### Near-miss assessment participants

In the case of maternal and neonatal near-misses, this project will involve working with health care facility personnel to identify both mothers and babies who had a life-threatening condition but survived. To be considered a “near-miss”, a woman will be screened using the “Maternal Near Miss Screening Tool” (Table 1), adapted from the World Health Organization (WHO) criteria for maternal near-misses [23] to better fit the low-resource setting of rural northern Ghana. A newborn must meet the criteria of a newly-developed “Neonatal Near-Miss Screening Tool” (Table 1) modeled after the WHO maternal criteria [23], again adapted for use in a low-resource setting. Participants will be mothers who survived their own near-miss or their baby’s near miss, unless the mother is unable to complete an interview or another family member is a more appropriate respondent (e.g. in the event the mother was not caring for the baby during its near-miss experience). The respondent must speak one of the following five languages: Mampruli, Sissali, Kasem, Nankam, or English; they must be healthy enough to carry on a conversation; and they must not be facing an imminent health crisis.

### Community Key Informants (CKIs)

Community Key Informants (CKIs) are volunteers who live within each community and serve as a liaison between the community and the health facilities. They are likely to know the most about traditional procedures related to maternal and neonatal health and can be an important source of knowledge as well as a source of entry into households [24]. CKIs will help field workers identify families who have experienced a maternal or neonatal death, and will ensure field workers understand local customs surrounding deaths including the customary grieving periods within each community that should be respected before participants are approached about participation in the project [24].

### Field workers

Field workers will be recruited from the four project districts to ensure an understanding of the local language and culture. The project team will rely upon the extensive training protocol in place for more than 25 years at NHRC’s Demographic Surveillance Site to ensure field workers are adequately prepared for project implementation. One of the project directors will work with the project implementation manager to conduct an intensive, 10-day training, including engaging the field workers in activities to simulate challenges in identifying individuals to interview, conducting mock interviews, and troubleshooting. Field workers will also be trained in geographic information system (GIS) data collection using hand-held tablets, including tracking their movements in the field, geocoding the location of critical land marks and structures, and remotely uploading data via the cellular network.

### Health care providers

Health care providers will assist with the identification and screening of “near-misses”. All providers in the project districts will be invited to participate in a project-specific provider training, in which the purpose of the project is described. Providers will be encouraged to notify field workers in the event of any maternal or neonatal death at the facility or that they hear of occurring before or after arrival at the facility. Providers will also be trained to complete a short screening form to identify any mothers or neonates who qualify as a “near-miss” (See Table 1). Health care providers will describe the project to any woman or guardian who qualifies to participate, and they will record the contact information of those who agree to participate. They will then notify the field worker to follow up for the interview at home. Field workers will check with providers at least once every two weeks, and providers will be encouraged to call the field workers when a “near-miss” is identified. Providers will be given phone credits to ensure their ability to call field workers.

### Community entry

**Fig. 2:**
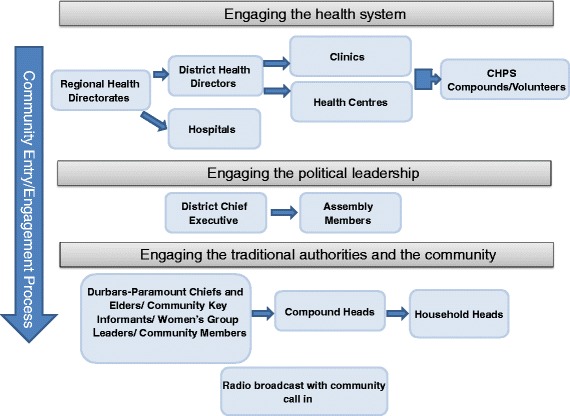
Process for entering communities

The formal community entry process is a critical part of project initiation, and the Navrongo team will draw upon their 25+ years of experience in this arena to ensure it is given proper attention [25]. The community entry process for PREMAND will be initiated by the Implementation Manager, building on the Navrongo Health and Demographic Surveillance Site (HDSS) channels already established in the Kassena-Nankana Districts (KNDs), including the HDSS field workers, community key informants and community leaders. A similar process will be used for the two project districts outside the existing purview of the HDSS: Sissala East in the Upper West Region, and East Mamprusi in the Northern Region. The political and traditional authority structures are relatively homogenous across the three study regions, making the lessons learned and processes used in the KNDs applicable in the other study districts (See Fig. 2). The entry process will be initiated through meetings with the Regional Health Directorates and District Health Directors. Members of the PREMAND team will describe the project to the District Director, answering any questions or concerns. The team will also meet with the District Chief Executive, again describing the project and offering the opportunity for questions and clarifications.

Next the PREMAND team will meet with Paramount Chiefs and Elders in each of the two districts, followed by health facility administrators and lastly, community volunteers. At each meeting, the team will describe PREMAND, introduce the field workers, discuss what the field workers will be doing as part of the project, and provide an opportunity for questions and clarifications. Community members, as well as CKIs and prominent community groups, will be invited to join the meetings. During this process, the team will suggest the possibility of working closely with the CKIs as an important source of knowledge as well as a source of entry into households for field workers.

The final component of community entry includes radio talk shows broadcast district-wide to introduce the community to the project. In districts where the PREMAND team leaders cannot communicate in the local language, the field staff will act as interpreters. After the introduction is completed, listeners will be encouraged to call in to ask questions, ensuring that the process is as interactive as possible for a mass media format. It will be explained that the project aims to generate information that can be used by communities and policy makers to help them better understand potential solutions to maternal and neonatal mortality.

### Participant recruitment

Field workers will coordinate with CKIs designated by the Navrongo Health Research Center and local health facilities to identify maternal and neonatal deaths and near-misses. Once cases are identified, field workers will visit the homes of the mothers and babies who died – or who came close to dying – and interview the individual who is in the best position to respond to questions about the events leading up to the death or near-miss event. All interviews will be conducted in the local languages.

### Participant consent

Trained field workers will describe the project to the participants and talk them through written consent forms that have been reviewed and approved as part of the ethical clearance granted by the institutional review boards at the Ghana Health Service, the Navrongo Health Research Centre, and the University of Michigan. The consent forms have been translated into one of four local languages: Mampruli, Sissali, Kasem, and Nankam.

The following elements of the consent form will be emphasized: 1) Participation is voluntary; 2) You may stop at any time; and 3) You may skip any question that you prefer not to answer. Literate respondents will be asked to sign a copy of the consent form. Non-literate respondents will be asked to thumbprint on the signature line. All respondents will be given a copy of the consent form with information about the study and the contact information for both the project team and the local IRB to keep for their records.

### Sample size calculation

This study is descriptive in nature and thus a calculation of sample size to show effects was not appropriate. We hope to identify and map all maternal and neonatal deaths and near-misses occurring in project districts during the study period. To generate estimates for project planning purposes, we first determined the total number of annual births across our districts. According to Ghana Health Service data, there are approximately 9000 births per year in these four districts. We multiplied that number by the national neonatal mortality rate of 29 per 1000 live births [26], to estimate there will be 261 neonatal deaths within our 1-year recruitment period. Note that the neonatal mortality rate is much higher than the maternal mortality rate in Ghana [27], and is therefore a better indicator of project resource needs. Absent firm data on the ratio of deaths to near-misses in northern Ghana, we borrowed an estimate for maternal near-misses in Ghana to suggest that for every death we would see at least 3 near-misses [27]. These numbers – while estimates – were within reason for our staff resources.

### Study instruments

This project involves the use of several study-specific instruments, as well as instruments that have been used in other contexts. Table 1 outlines and describes all PREMAND screening tools, and Table 2 outlines and describes PREMAND interview tools.

**Table 2:** PREMAND project interview tools

Interview tools	Description
Health Facility Assessment tool	This is a study-specific instrument that asks basic information about each health care facility, including such things as the number and type of providers, the provision of antenatal care, delivery care, and post-natal care, and the ability to carry out various signal functions for basic andcomprehensive emergency obstetric care [31]. It also goes beyond traditional facility assessments by including questions on the frequency of various practices. This instrument will be completed by field staff as they visit each facility within the four project districts.
Maternal Verbal and Social Autopsy (MVASA)	The Verbal Autopsy portion of this tool is based upon the existing “Standard Verbal Autopsy Questionnaire for Adolescent and Adult Deaths (12 years and over)” in use at the Navrongo Health Research Centre. A few questions were added to ensure all relevant clinical data were collected for the purposes of this project. The Social Autopsy portion of the tool combines the INDEPTH Social Autopsy Tool [16], elements of the Child Health And Epidemiology Research Group (CHERG)’s Social Autopsy Tool (Kalter et al., 2011), and additional items addressing social and cultural factors related to health seeking, such as social support, community norms, and attitudes toward traditional providers.
Neonatal Verbal and Social Autopsy (NVASA)	The Verbal Autopsy portion of this tool closely mirrors the existing “Standard Verbal Autopsy Questionnaire for Neonatal Deaths (0-27 days old)” in use at the Navrongo Health Research Centre. A few questions were added to ensure all relevant clinical data were collected for the purposes of this project. The Social Autopsy portion of this tool combines the INDEPTH Social Autopsy Tool, the Child Health And Epidemiology Research Group (CHERG)’s Social Autopsy Tool [16, 17], and additional items addressing social and cultural factors related to health seeking, such as social support, community norms, and attitudes toward traditional providers.
Maternal Near-Miss Sociocultural Assessment (MSCA)	This interview tool is a study-specific survey instrument that is loosely based upon the INDEPTH Social Autopsy Tool, elements of the Child Health And Epidemiology Research Group (CHERG)’s Social Autopsy Tool [16, 17], and additional items from the existing verbal autopsy instruments in use at the Navrongo Health Research Centre. The interview tool is designed to elicit input on the social and cultural factors associated with near-misses among mothers. It also includes selected items addressing social and cultural factors related to health seeking, such as social support, community norms, and attitudes toward traditional providers.
Neonatal Near-Miss Sociocultural Assessment (NSCA)	This interview tool is a study-specific survey instrument that is loosely based upon the INDEPTH Social Autopsy Tool, the Child Health And Epidemiology Research Group (CHERG)’s Social Autopsy Tool [16, 17] and additional items from the existing verbal autopsy instruments in use at the Navrongo Health Research Centre. The interview tool is designed to elicit input on the social and cultural factors associated with near-misses among neonates. It also includes selected items addressing social and cultural factors related to health seeking, such as social support, community norms, and attitudes toward traditional providers, among other things.

### Data collection

PREMAND will collect quantitative health data, qualitative health data, and geographic data. Quantitative information will be recorded using pre-loaded surveys on hand-held, GPS-enabled Google Nexus Tablets (see Table 2), allowing the interviewers to ask questions and immediately enter the data. The SurveyCTO platform (Cambridge, Massachusetts) will be used to support secure data collection and storage. Qualitative information will be prompted using a pre-loaded survey on the data collection tablets, but all interactions will be recorded using a digital audio recorder. All interviews will be conducted in the local languages, audiotaped, and transcribed into English by the interviewers themselves to ensure accuracy. Interviews will be reviewed by the project field coordinator and implementation coordinator to ensure adherence to the interview guide, sufficient probing for additional information, and acceptable transcription. Geographic data will be collected in latitude/longitude format using the GPS-enabled tablets. The locations of health facilities, traditional healer compounds, chief compounds, local markets, dams, and roads will be collected by field workers and combined with existing maps of the project districts.

### Data analysis

#### Quantitative health data

Quantitative data will include such variables as cause of death (COD) or near-miss, sociodemographic factors of the mother or the baby, and care-seeking related variables. COD will be determined using NHRC’s standard verbal autopsy protocol, using a panel of three physicians who independently review responses to the verbal autopsy portion of the surveys. If there is agreement of at least two physicians, a COD will be determined. If there is a disagreement amongst all three physicians, the VA responses will be sent to two further physicians for review and COD determination. In cases where consensus cannot be reached amongst the second panel of physicians, the COD will be coded as “undetermined”. Cause of near-misses will be established through the use of the near-miss screening tools, which define cases based upon symptoms and clinical management. The near-miss screening tools also include a section for health care providers to mark the most likely cause of the near-miss, as well as any suspected underlying or contributing causes.

Statistical analysis will be conducted using Stata 13.1 (Statacorp, College Station, Texas). Descriptive statistics showing means and standard deviations will be conducted for normally distributed data while the median and inter quartile ranges will be reported for data that are not normally distributed. Bivariate and multivariate analysis will be performed to determine factors that are associated with mortality or near-misses.

#### Qualitative health data

All qualitative data will be transcribed into English, with any translation uncertainties discussed amongst the research team and determined via consensus. Transcripts will be entered into NVivo 10.0 (QSR International, Victoria, Australia), qualitative analysis software. After reading and re-reading the transcripts, the project team will work together to generate a preliminary coding structure to guide the coding of all qualitative interviews. A detailed codebook will be created, including detailed descriptions of what is included and excluded from each code, as well as the hierarchy of codes. The codebook will then be used to guide the qualitative coding.

Qualitative coding will be conducted by at least two team members, bringing in a third in the case of discrepancies in codes. Coders will meet weekly to discuss the analysis process, identify potential new codes, and revisit coding boundaries. Coders will also participate in routine meetings with the Implementation Manager and field workers to discuss such things as:

- Main themes emerging from the interviews
- The degree to which interview themes or issues are repeating
- The emergence of conflicting findings
- Information gaps for further follow-up
- Aspects of the interview process that might need improvement
- Data collectors’ perspectives on the data collection activities and their observations in assorted community settings.

#### Geographic data

The location of all deaths and near-misses will be geo-tagged and placed on a map (blurred to obscure exact location for non-study personnel), with linked data available for visual analysis of patterns and observable trends. Distance between respondents’ homes and the facilities visited will be calculated and treated as a potential predictor variable in multivariate regression analyses comparing deaths and near-misses. Analysis of spatial clustering will be conducted to determine if there are specific locations within the districts where deaths or near-misses cluster.

### Geographic mapping

To create detailed maps for each of the four project districts, PREMAND health indicator data will be combined with PREMAND geographic data and supplemented by other sources of geographic data from northern Ghana. PREMAND visualizations will include background layers with the option to include health facilities, roads and locations of traditional healers to better illustrate the local context. Maps will be populated with variables selected in the data analysis stage in order to visualize and further examine the locational relationship between different pieces of project data. The range of variables to be compared may include: cause of death/near miss; household location; delivery location; delivery assistance (traditional birth attendant/nurse/midwife/medical doctor); type of delivery; traditional care sought; number of providers seen; maternal age at time of near-miss/death; infant age at time of near-miss; infant gender; time of day of birth; umbilical cord care; insurance status; maternal education; religion; household wealth; social support; and community norms. Examples of potential relationships between variables to be visualized could include:

- *Location of sepsis-related infant deaths* relative to *umbilical cord care practices*; or
- *Care seeking patterns* relative to *community norms regarding traditional medicine;*
- *Locations of maternal deaths or near-misses* relative to *locations of health facilities.*

Maps will also incorporate qualitative data and/or personal narratives to illustrate the stories behind the data and emphasize the experience of PREMAND respondents from the local communities.

Project maps will be created in two forms: a customized, password-protected portal will allow relevant stakeholders to interact with the data online, and printed maps will be presented locally in the four project districts through community meetings. A custom mapping application will be built and populated that will allow stakeholders to visually explore the occurrences and correlates of deaths and near-misses. The customized portal will feature geographic base layers of the project districts, and will allow selected users to combine multiple types of PREMAND health data to visually explore the relationship between different variables. This interactive web application will be tailored to the needs of government health officials and policy leaders. The portal will also give them the ability to compare regions as well as zoom in where necessary to better understand the dynamics taking place at the community-level.

Maps will also be designed for stakeholders such as chiefs, health facility staff, mothers, and traditional healers. Project findings will be prepared as large-scale, printed paper maps and presented to the communities in their local languages. Maps will also feature the relationships between different geographic and health indicator data, but variables will be pre-selected by the PREMAND team based on the patterns in the data found during preliminary analysis that are likely to be most useful for members of the individual communities. Because community members in many of the project districts may be illiterate, the PREMAND team will work with field workers and local community volunteers to determine the best presentation style for different communities. Similarly, printed maps presented at the community-level may rely more heavily on images, while maps available through the online portal may contain a greater proportion of text, depending on the needs and interests of the different audiences.

### Community engagement/innovation sites

The PREMAND team will use the mapping application to identify observable patterns in each of the five project zones. The project team will consult with district health officers and its technical advisor to select one community in each district for the implementation of community-driven programming tailored specifically to address the local contributing factors influencing maternal and neonatal health in their communities. The team will then approach community leaders and chiefs in each of the designated communities to discuss project findings and gauge interest in participating as a pilot site. It is important to the team that this buy-in comes from the leadership of the communities, as any health programming in the area only makes sense if it will serve them, and any intervention should be invited. For this reason, the team will identify more than one community per project zone, so that others can be approached in the event that the first communities selected are not interested in participating.

Once the final list is determined, these innovation sites will receive a grant of up to $2000 administered by the NHRC to be used to respond to local-level factors contributing to maternal and neonatal mortality in the way that community stakeholders deem appropriate. The amount of $2000 was selected as an appropriate amount for the community pilot grants, as it is sufficient to support grassroots initiatives but not so large as to create a windfall of funding that would disturb the current way of life in the selected communities. Smaller grants will encourage communities to identify low-cost solutions that can endure beyond the life of the project.

The recipients of the grants will be determined through consultations with community leaders and District Health Management Teams, and will likely be a particular stakeholder group. Potential low-cost solutions could include such things as generating a volunteer pool of “on-call” drivers willing to transport pregnant women or mothers with their newborns to the hospital or health center, or health information campaigns targeted at male household heads, in communities where their permission is required for women to seek clinic-based care.

Once community buy-in is achieved and community selections have been finalized in each project district, the PREMAND team will work with community leaders to design local programming. Through careful facilitation by project staff, each community will be supported in 4–6 months of activities designed to take steps to address their specific community-level challenges that contribute to maternal and neonatal mortality.

At each innovation site, key stakeholders will include chiefs, community leaders, community health providers, and other stakeholders that are likely to vary by location. In addition, each innovation site will be located within the purview of a District Health Management Team, which will also be seen as a key stakeholder. Using the principles of participatory action planning, the project team intends to engage as many key stakeholders as possible from all of these groups in an iterative process of developing, planning, and implementing innovation site activities. While the implementation team will serve as the facilitator for such efforts, activities will ultimately be planned and implemented by a coalition of key stakeholders from each innovation site.

Program planning will commence with the chiefs who were initially consulted in the identification phase, as well as community key informants, and district health officers. These stakeholders will be briefed on the findings of the social autopsy and near-miss study in their community and surrounding area, and given paper copies of the maps visualizing these findings, so that information gathered in Years 1 and 2 of the project can be effectively translated into locally-relevant programming. Together, the group will brainstorm programming ideas and community stakeholders will determine the final activities and approach.

While the exact topics of each community’s focus will be contingent on the social autopsy findings, the correlations captured in the visualizations, and community-generated decisions, potential programming could include such things as:

- Clinic-specific interventions targeting low-performing clinics;
- Community-driven transportation solutions to ensure prompt access to care; or
- Integration of non-traditional stakeholders such as grandmothers, husbands, and compound heads into maternal and neonatal health programming.

The project team will assist in coordinating stakeholders and organizing meetings. Where it is deemed helpful, the team will use its connections to GHS to contribute venue space for activities. The implementation manager will assist in managing pilot grants and purchasing materials or supporting meeting expenses for the community out of their $2000 innovation site budget. Where relevant, the team will contribute its technical expertise in medicine and public health to the community programming. This may include giving a presentation at a community event, or advising on the language used in posters or pamphlets, depending on the priorities each site identifies.

The project team will maintain ongoing contact with the community leadership throughout the pilot grant period and provide assistance as needed. While a rigorous outcomes assessment of each pilot grant is beyond the funding capability of PREMAND, the project team will work to conduct formative and process-based evaluation, as well as documenting successes, challenges, and lessons learned through each locally-driven program. All documentation will be used to develop a report containing case studies from all five pilot sites that will be distributed to USAID, GHS, and the Ministry of Health.

### Ethical approval

The research and ethical review boards at the Navrongo Health Research Centre (NHRCIRB194), the Ghana Health Service (GHS-ERC: 05/01/15) and the University of Michigan (HUM00093372) reviewed the protocol and all instruments associated with the project and either approved (NHRC, GHS) or exempted (UM) the project from further review. Additional file 1-3 illustrate documentation of IRB approvals.

### Ethical considerations

There are several ethical considerations unique to a project like this one. First, the sensitive nature of social autopsy and near-miss research means that participants may not be comfortable discussing their health or the health of their family members, especially in the project districts with less exposure to research. To mitigate this risk, the project created a detailed “community entry protocol” to ensure thoughtful and strategic community entry that takes into account the social hierarchies and preferences of each community. Moreover, the informed consent process will provide potential participants detailed information about the study and will make clear that participation is voluntary and can be ended at any time. Second, participants may be concerned that the collection of geographic data will identify individual households where deaths or near-misses occur. To protect the privacy of respondents, project maps will blur the locational data so that exact locations cannot be identified. The project team will use polygon data to mark the approximate area without depicting the locations at the household level. Finally, there is the potential for communities that are identified as worthy of further intervention as a result of PREMAND to feel stigmatized. Communities may be hesitant to participate as “innovation sites,” given that sites will be identified based on high levels of deaths and near-misses and local social and cultural practices that contribute to these adverse outcomes. Project leadership will meet personally with community leaders to present our project findings and gauge their interest in participating as a project innovation site. Findings will be presented in a way that emphasize improvements seen in Ghana over previous years, and the positive nature of having high numbers of ‘near-misses’ – as opposed to deaths. Findings will also emphasize the possibility to move along the continuum, from mortality to near-misses toward a community with healthier mothers and infants. Only communities that demonstrate an interest in the project findings will be invited to participate in the innovation site activities.

### Project timeline

PREMAND is a three-year project, and Table 3 illustrates the project timeline.

**Table 3:**
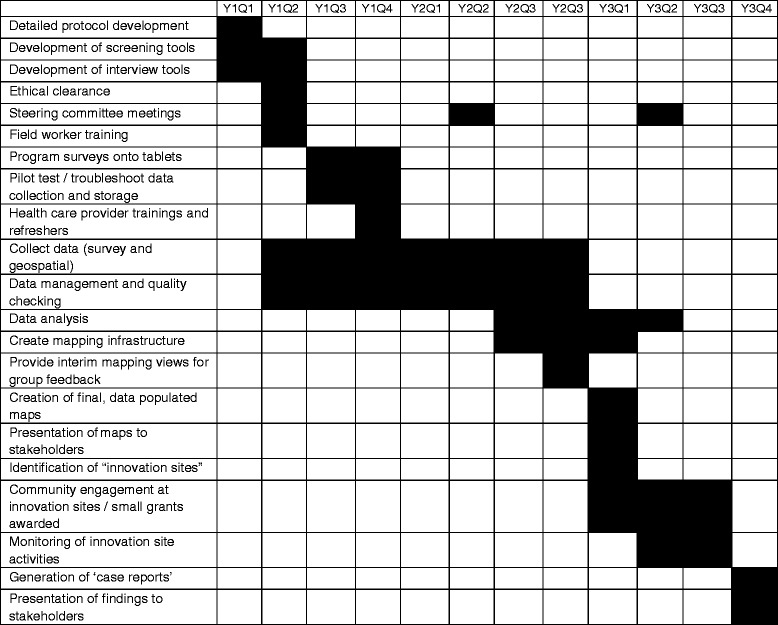
PREMAND project timeline

## Discussion

PREMAND uses an innovative, multifaceted approach to better understand and address neonatal and maternal morbidity and mortality in northern Ghana. We believe PREMAND will yield important information about the social and cultural factors that contribute to maternal and neonatal deaths and near-misses in the project regions. PREMAND is the first project we are aware of to both geolocate deaths and near-misses but also to apply a sociocultural lens through the use of social autopsies for mothers and babies who died and sociocultural audits for those who nearly died but ultimately survived. We hope these sociocultural assessments will expand our understanding of the factors that distinguish between deaths and near-misses in low-resource settings. The mapping component of PREMAND will result in detailed maps of the project districts that illustrate areas with high concentrations of adverse outcomes and the patterns associated with these events. We believe this translation of highly technical research data into an easily-understandable map will make this information approachable and usable by decision-makers at all levels. The final phase of the project will result in “innovation sites” that utilize these maps to create locally-driven programming in communities with high maternal or neonatal health risks. Innovation sites will provide insights on a variety of prevention approaches and their efficacy and will demonstrate a community-driven program design process that puts communities in charge of designing locally-relevant solutions. Donors and government leaders will receive comprehensive information outlining the solutions developed and the lessons learned from these community-driven approaches.

While we are optimistic about the potential impact of PREMAND, we acknowledge that there are both limitations to this approach, as well as challenges to its implementation. One limitation is that the project is limited to four districts in northern Ghana, as opposed to covering a wider area where a greater number of deaths could be expected. Given the sparse, rural population in northern Ghana, even those districts with relatively high mortality rates will not see a large number of deaths in a calendar year. While this is good news for northern Ghana, it creates limitations to our ability to conduct meaningful statistical analyses related to mortality. We hope to have addressed this issue by recruiting near-misses as well, thus exploring the issues that perhaps distinguish between deaths and near-misses, or are associated with certain causes of deaths and near-misses. We also believe that visual analysis may provide more meaningful information for local stakeholders than statistical analysis – if there are trends or associations visible to the naked eye on a map, it is likely to hold more value locally than the results of a statistical test. One notable challenge to this approach is the significant amount of time required: first, to develop valid tools to measure sociocultural variables appropriate to the setting; second, to ensure the functioning of an electronic data collection system in a region with intermittent power, limited wifi, and spotty cellular coverage; and third, to effectively and appropriately enter communities spread across four disparate districts. The PREMAND timeline is aggressive, yet we believe we can accomplish the project aims within the timeframe allocated.

In sum, PREMAND is an innovative, three-phase project in rural northern Ghana that we hope will shed light on the social and cultural factors contributing to maternal and neonatal morbidity and mortality through the use of geospatial visualization and locally-driven community interventions.

## Additional files

Additional file 1: GHS IRB approval. (PDF 536 kb) Additional file 2: UM IRB approval. (PDF 67 kb) Additional file 3: NHRC IRB Approval. (PDF 755 kb)

## Abbreviations

CKIs: Community Key Informants
COD: cause of death
GHS: Ghana Health Service
GIS: Geographic Information System
GPS: Global Positioning System
HDSS: Health and Demographic Surveillance Site
IRB: Institutional Review Board
KNDs: Kassena-Nankana Districts (KNDs)
NHRC: Navrongo Health Research Centre
PREMAND: PREventing Maternal And Neonatal Death
WHO: World Health Organization
