# Supplementary material for: PREventing Maternal And Neonatal Deaths (PREMAND): a study protocol for examining social and cultural factors contributing to infant and maternal deaths and near-misses in rural northern Ghana
Source: Reprod Health. 2016 Mar 9;13:20. doi: 10.1186/s12978-016-0142-z (PMC4784316; doi:10.1186/s12978-016-0142-z)
Supplement: Additional file 1: — GHS IRB approval. (PDF 536 kb) [file 12978_2016_142_MOESM1_ESM.pdf]

GHANA HEALTH SERVICE ETHICS REVIEW COMMITTEE

In case of reply the
number and date of this
Letter should be quoted.

My Ref. :GHS-ERC: 3
Your Ref. No.

Research & Development Division
Ghana Health Service
P. O. Box MB 190
Accra
Tel: +233-302-681109
Fax + 233-302-685424
Email: Hannah.
Frimpong@ghsmail.org

2nd April, 2015

Dr. John E. Williams
Navrongo Health Research Centre
Ghana Health Service
P. O. Box 114
Navrongo

ETHICAL APPROVAL - ID NO: GHS-ERC: 05/01/15

The Ghana Health Service Ethics Review Committee has reviewed and given approval for the implementation of your Study Protocol titled:

"Preventing Maternal and Neonatal Mortality in Rural Northern Ghana"

This approval requires that you inform the Ethical Review Committee (ERC) when the study begins and provide Mid-term reports of the study to the Ethical Review Committee (ERC) for continuous review. The ERC may observe or cause to be observed procedures and records of the study during and after implementation.

Please note that any modification without ERC approval is rendered invalid.

You are also required to report all serious adverse events related to this study to the ERC within seven days verbally and fourteen days in writing.

You are requested to submit a final report on the study to assure the ERC that the project was implemented as per approved protocol. You are also to inform the ERC and your sponsor before any publication of the research findings.

Please note that this approval is given for a period of 12 months, beginning April 2nd 2015 to April 1st 2016.

However, you are required to request for renewal of your study if it lasts for more than 12 months.

Please always quote the protocol identification number in all future correspondence in relation to this approved protocol

SIGNED...

DR. CYNTHIA BANNERMAN
(GHS-ERC CHAIRPERSON)

Cc: The Director, Research & Development Division, Ghana Health Service, Accra
