# Supplementary material for: PREventing Maternal And Neonatal Deaths (PREMAND): a study protocol for examining social and cultural factors contributing to infant and maternal deaths and near-misses in rural northern Ghana
Source: Reprod Health. 2016 Mar 9;13:20. doi: 10.1186/s12978-016-0142-z (PMC4784316; doi:10.1186/s12978-016-0142-z)
Supplement: Additional file 2: — UM IRB approval. (PDF 67 kb) [file 12978_2016_142_MOESM2_ESM.pdf]

Health Sciences and Behavioral Sciences Institutional Review Board • 540 East Liberty Street, Suite 202, Ann Arbor, MI 48104-2210 • phone (734) 936-0933 • fax (734) 998-9171 • irbhsbs@umich.edu

To: Cheryl Moyer

From:

Thad Polk

Cc:

Cheryl Moyer

Subject: Notice of Determination of "Not Regulated" Status for [HUM00093372]

SUBMISSION INFORMATION:
Title: Preventing Maternal and Neonatal Mortality in Rural Northern Ghana
Full Study Title (if applicable):
Study eResearch ID: HUM00093372
Date of this Notification from IRB: 9/15/2014
Date of IRB Not Regulated Determination: 9/15/2014

IRB NOT REGULATED STATUS:

Category	Outcome Letter Text
**Quality Assurance and Quality Improvement Activities - Other**	Based on the information provided, the proposed study does not fit the definition of human subjects research requiring IRB approval (per 45 CFR 46, 21 CFR 56 and UM policy). Although the results of your project may be published, program evaluations, self-assessment of programs or business practices, and other quality improvement projects do not require IRB review because in these cases, it is the activities rather than humans subjects that are the objects of the study.

Thad A. Polk

Thad Polk
Chair, IRB HSBS
