# Supplementary material for: PREventing Maternal And Neonatal Deaths (PREMAND): a study protocol for examining social and cultural factors contributing to infant and maternal deaths and near-misses in rural northern Ghana
Source: Reprod Health. 2016 Mar 9;13:20. doi: 10.1186/s12978-016-0142-z (PMC4784316; doi:10.1186/s12978-016-0142-z)
Supplement: Additional file 3: — NHRC IRB Approval. (PDF 755 kb) [file 12978_2016_142_MOESM3_ESM.pdf]

In case of reply the number and date of this letter should be quoted.

My Ref. App/PreMAND /02/2015
Your Ref:

Navrongo Health Research Centre
Institutional Review Board
Ghana Health Service
P. O. Box 114
Navrongo, Ghana
Tel/Fax: +233-3821-22348
Email: irb@navrongo.mimcom.org

12th February, 2015

Dr. John Williams
Navrongo Health Research Centre
Ghana Health Service
P.O. Box 114
Navrongo, UER

ETHICS APPROVAL ID: NHRCIRB194

Dear Dr. Williams,

Approval of study titled *Preventing Maternal and Neonatal Mortality in Rural Northern Ghana*

Following your satisfactory address of the concerns raised by the NHRCIRB during its full Board review of the above-mentioned protocol, the Board is pleased to grant you approval.

The documents that were reviewed and approved include the following:

- Protocol submission form
- Study protocol version 2.0 dated 16/01/2015
- Questionnaire for the study version 2.0 dated 16/01/2015
- Consent forms – English Version 2.0 dated 16/01/2015 for Adult
- Consent forms – Kasem Version 2.0 dated 16/01/2015for Adult
- Consent forms – Nankam Version 2.0 dated 16/01/2015 for Adult
- Consent forms – Sissali Version 2.0 dated 16/01/2015 for Adult
- Consent forms – Mampruli Version 2.0 dated 16/01/2015 for Adult
- Screening tools Version 2.0 dated 16/01/2015
- Curriculum Vitae of Investigators

Please note that any amendment to this approved protocol must receive ethical clearance from the NHRCIRB before its implementation.

Should you require a renewal of your approval, a progress report should be submitted two (2) months before the expiration date.

You are also to note that this approval expires on 11th February, 2016.

The Board wishes you all the best in this study.

Sincerely,

Dr. (Mrs.) Nana Akosua Ansah
(Vice Chair, NHRCIRB)

Cc: The Director
NHRC, Navrongo
